# Time After Time: Attachment Orientations and Impression Formation in Initial and Longer-Term Team Interactions

**DOI:** 10.3389/fpsyg.2022.882162

**Published:** 2022-05-30

**Authors:** Dritjon Gruda, Raul Antonio Berrios, Konstantinos G. Kafetsios, Jim Allen McCleskey

**Affiliations:** ^1^School of Business, Maynooth University, Maynooth, Ireland; ^2^Facultad de Administración y Economía, Universidad de Santiago, Santiago, Chile; ^3^School of Fine Arts, Aristotle University of Thessaloniki, Thessaloniki, Greece; ^4^Katedra Psychologie, Palacký University, Olomuc, Czechia; ^5^Western Governors University, Salt Lake City, UT, United States

**Keywords:** attachment theory, team, social interactions, experiment, diary study

## Abstract

If securely attached individuals typically exhibit more desirable attributes, can insecure individuals be perceived positively when working in teams despite their interpersonal disadvantages? In an exploratory study, using both a vignette based experimental research design (*n* = 636) and a round-robin study of professionals working on a team task for nine consecutive weeks (*k* = 648), we examined the evolving impressions of insecurely attached individuals over time. We find that while anxiously attached individuals are perceived more positively in initial interactions, this initial positive effect for anxious attachment disappeared over time as individuals within teams gained more relational knowledge about their team members. We also found a stable and negative effect of avoidant attachment. We discuss possible reasons for the temporal underpinnings of this effect and compare our findings to previous literature.

## Introduction

Attachment theory counts among the most influential theoretical models with an observed fierce interest in its application in work and organizational contexts (see Yip et al., 2018; Gruda and Kafetsios, 2020). As workplace interactions are relational, attachment theory is, therefore, an especially effective framework for understanding interpersonal work experience in both horizontal (Yip et al., 2018) and hierarchical (Mayseless and Popper, 2019) interactions at work. Much of this effort to understand interpersonal attachment dynamics at work has erred toward a behavioral view of attachment dynamics at work at the expense of perceptual aspects. Yet, existing related evidence from attachment theory in leader-follower interactions (Kafetsios et al., 2014; Kafetsios and Gruda, 2018) point to the key role of attachment-related perceptions in organizational contexts.

The present manuscript presents results from a cross-sectional experiment (Study 1) and a longitudinal team study (Study 2) designed to examine initial and evolving perceptions of anxious and avoidant attachment orientations in team interactions. By doing so, this study makes several contributions. First, we examine the role of attachment schema perception under different levels of acquainship in social relationships: early and later stages of relationships at workin team member dyads. Secondly, given the longitudinal design of Study 2, we also showcase that team members’ perceptions of target teammates’ attachment orientations vary across specific partners and over time.

### Attachment Theory and Interactions at Work

Varying on the avoidance and anxiety dimensions, attachment orientations involve mental working models of significant others, the self, and the relationship between the self and significant others that results from interactive experiences with others. Anxious attachment is defined by a corresponding working model which dictates that others are viewed more positively than the self. This typically leads individuals to experience a strong need for intimacy and increased proximity and closeness to important others (Yip et al., 2018). The corresponding working model of avoidant attachment involves a negative view of and distance from others, with an emphasis and focus on the self and deactivation of the attachment system by suppressing and limiting access to emotional memories and thoughts (Mikulincer and Shaver, 2007).

Work on attachment and contextual work performance has shown that anxious attachment is related to lower levels of helping behavior at work (Geller and Bamberger, 2009). Avoidant persons, on the other hand, have difficulty functioning optimally in organizational environments and suffer decreased effectiveness (Richards and Schat, 2011), which should make such individuals less desirable as team members. Yet, some research suggests that, at least in the case of leader-follower interactions, leader avoidance can be associated with some favorable follower work outcomes and leader perceptions in groups of followers characterized by higher interdependent schemas (Kafetsios and Gruda, 2018; Gruda and Kafetsios, 2020). Finally, securely attached individuals, those who score low on anxious and avoidant attachment, are more sought after for leader and team membership (Berson et al., 2006).

In sum, we examine whether insecure individuals could be perceived positively when working in teams despite the association between insecure attachment orientation and interpersonal disadvantages. In addition, we test whether a possible positive perception of insecure attachment orientations is merely fleeting or long-lasting. Building on previous research from relationships science (Brumbaugh and Fraley, 2010), we argue that a high degree of anxious attachment may be of benefit to the perception of individuals in initial interactions during which such individuals may be perceived as desirable team members who elicit positive emotions in others. Similarly, we argue that the perceptual effects of avoidant attachment are more uniformly negative across initial and subsequent work interactions.

### Anxious Attachment in Initial Interactions

When individuals’ attachment system is activated, secondary, insecure, strategies of avoidance and anxiety come into play. Anxious attached individuals are likely to engage in hyperactivating behavior, which drives them to increase proximity seeking. For example, anxious individuals engage in hypervigilance and crave to please and form connections with others (Geller and Bamberger, 2009). This would explain why, at least in initial romantic interactions, anxious persons tend to be perceived as caring, attentive, and feedback-seeking (Brumbaugh and Fraley, 2010). This is in stark contrast to evidence in established relationships, that consistently demonstrates that anxious individuals are likely to be perceived negatively by others (Mikulincer and Shaver, 2007). We, therefore, expected that the coping strategy of anxious individuals (i.e., increased closeness and a strong desire to connect with and rely on others) may be perceived positively during dyadic interactions at the very early stages of a relationship.

What does this mean for interactions at the workplace? In initial interactions, such as joining a new work team, individuals tend to experience high levels of stress and are motivated to present themselves in the best light possible. To do so successfully and given situational demands, we argue that anxious individuals’ over-attentive and hypervigilant behavior is likely to be perceived as caring and warm, in turn fostering positive feelings from their (dyadic) team members (Brumbaugh and Fraley, 2010). That is because anxious persons are more likely to want to conform with, and please others to fulfill their own attachment needs (Davidovitz et al., 2007; Game et al., 2016). Therefore, we hypothesize the following:

*H1*: *In initial interactions, individuals’ anxious attachment will elicit more positive emotions and positive perceptions in others.*

### Avoidant Attachment in Initial Interactions

Deactivation of the attachment system is associated with a generalized working model marked as an insecure avoidant orientation. Avoidant individuals have lower positive perceptions for experiencing rewarding social interactions in more intimate relationships (e.g., Kafetsios and Nezlek, 2002). Avoidant persons are also unlikely to adopt an approach-orientated behavior in initial interaction unless triggered by the relational interaction partner. For example, Spielmann et al. (2013) found that when an opportunity for an intimate relationship was clear (an existing relationship versus an ex-partner, an imagined future relationship vs. an imagined reunion, a responsive or non-responsive prospective date), avoidant participants perceived lower opportunity for intimacy.

However, where there is limited information regarding avoidance in general social interactions; indeed, partly as a strategy to preserve distance, avoidant individuals are motivated to reduce unpleasantness in interactions overall (Brumbaugh and Fraley, 2010). From a developmental-behavioral perspective, avoidance is considered as a learned reaction to consistently preserve autonomy in interpersonal relations as a result of predictably unresponsive caregiving (Strand, 2020). Hence, in initial work interactions - such as starting in a new team – avoidant individuals likely would aim to improve their self-perception by maintaining a negative view of others. We hypothesize the following:

*H2*: *In initial interactions, individuals’ avoidant attachment will elicit less positive emotions and less positive perceptions in others.*

### Relationship Tenure

We expect that, over time, high levels of anxious attachment to others would likely harm relationship quality and functioning. Most previous studies have found a negative relationship between anxious attachment and relationship satisfaction and interaction partner perception (e.g., Kafetsios et al., 2014). According to Brumbaugh and Fraley (2010, p. 602), anxious individuals might initially appear to exhibit positive relational features, yet their insecurity likely leads to “considerable relational problems down the road”. Conversely, given that avoidant persons are socialized in relationships where there is a high level of predictability of affective signals from important others, accordingly, we hypothesized the following:

*H3: Over time, anxious individuals will fail to sustain initial positive emotions in others (H3a) while avoidant individuals will maintain initial negative perceptions in others (H3b).*

## Overview of Studies

We tested our hypotheses across two studies. In Study 1, we examined the impact of anxious and avoidant attachment on perceptions of initial interactions, with no prior attachment-related information on which judgments about the other person could be based (Mikulincer and Shaver, 2007). In such situations, scholars are likely to “detect the purest effects of chronic working models on relational behavior” (Mikulincer and Shaver, 2007, p. 286). Given that first impressions are also influenced by perceivers and given the pervasiveness of attachment as socio-cognitive schemas (Turan, 2016) we examined whether descriptions of anxious or avoidant insecure patterns are readily recognized. To do so, we set up a vignette study (Study 1), designed to mimic the evaluation of potential job candidates displaying either anxious or avoidant attachment impressions and compared these to a control condition.

In Study 2, we examine the impact of anxious and avoidant attachment on evaluations of trust and positive and negative affect as well as conflict in developing teams. We collected repeated interaction data over time between team members working toward a common goal, using a round-robin design (Kenny and Lavoie, 1984; Kenny, 1994).

## Study 1

We set up an online experiment using descriptive job candidate vignettes. Our final sample consisted of 636 United States (45.44% female) participants. Participants were recruited via Amazon’s Mechanical Turk to take part in this experiment. Participants were between 19 and 73 years of age (*M* = 40.15, *SD* = 11.78), with an average work experience of 18.27 years.

After providing consent, participants were informed that job candidates already had been pre-screened, and they were tasked with interviewing and evaluating one particular job candidate, Mark Smith. All participants were shown the same description of Mark. The description included information on Mark’s past work experience and educational background. Following this initial description of Mark, participants were provided with a brief scenario description, in which the participant was the interviewer in the room with Mark:

*“Interviewer: Thank you for coming in Mark. We have discussed your previous experience with the rest of the management team already. So far, we like what we see. Now, since the position you applied for requires a close relationship and exchange with your direct supervisor and team, we are particularly interested in hearing more about your expectations regarding that relationship. Could you tell us a bit more about that?”*

Participants were then randomly shown one of three possible answers, either an anxious attachment (ANX), an avoidant attachment (AVOID), or a control condition. All three vignettes were identical in sentence structure and word count, based on 115 words and 6 sentences.

The ANX and AVOID vignette was composed based on an established attachment scale specifically designed for the workplace (Game, 2008). Both attachment vignettes were purposefully designed to focus on the direct supervisor relationship. The ANX vignette included phrases such as “My previous supervisor and I became good friends and I hope that will be the case here as well.” The AVOID condition included sentences such as “My previous supervisor and I had a formal working relationship and I hope that to be the case here as well.” The control condition was designed to focus more on relationships with the overall team. It included sentences such as “My previous team and I got along quite well, and I hope that will be the case here as well.” Full vignettes are provided in Table 1.

**TABLE 1:** Description of vignette conditions (Study 1).

Condition name	Condition text	Word count
Anxious attachment condition	**Mark:** Thank you for inviting me in, pleasure to be here. To answer your question, well, I like to work closely with my direct supervisor. I believe that getting to know my supervisor as a person builds a strong team dynamic. I’m also happy to spend additional time with my supervisor to get the job done. I mean, I always try to help out whenever I can. I’m sure my supervisor would do the same for me as well. My previous supervisor and I became good friends and I hope that will be the case here as well. After all, the most important thing for me is to be the best team member I can.	115
Avoidant attachment condition	**Mark:** Thank you for inviting me in, pleasure to be here. To answer your question, well, I like to have a functional and professional relationship with my supervisor. Hence, unless required, I try to avoid having to go to my supervisor for help. I’m sure I could spend additional time at the office to get the job done, if needed. I mean, I like to solve problems on my own. And I’m sure my supervisor would expect me to work independently. My previous supervisor and I had a formal working relationship and I hope that to be the case here as well. After all, the most important thing for me is to get the work done.	115
Control condition	**Mark:** Thank you for inviting me, pleasure to be here. To answer your question, well, I like to have a good relationship with others. I believe that getting to know my team a bit better builds a strong team dynamic. I’m also quite willing to spend additional time at the office when necessary. I mean, I just try to be useful whenever I can. I’m sure others here would do the same for me as well. My previous team and I got along quite well and I hope that will be the case here as well. After all, the most important thing in this job is to get along well with the rest of the team.	116

Thirdly, participants evaluated the presented job candidate description on positive and negative affect (PANAS). In addition, we asked participants to indicate whether (and to what degree) they would expect conflict while working with Mark. As a final evaluation question, participants were asked to indicate whether, based on the description alone, they would like to work with Mark.

Subsequently, we asked participants to complete several individual differences scales. Measures consisted of global attachment (Fraley et al., 2015) and Big Five personality (Goldberg et al., 2006). A manipulation check was conducted to examine whether participants remembered the description of the fictional character, Mark, correctly, i.e., if they were paying attention during the experiment. Following this additional check, participants completed demographic measures. Finally, participants were fully debriefed and thanked for their help.

### Measures

#### Positive and Negative Affect

Positive and negative affect were measured using the Short Form of the Positive and Negative Affect Schedule (I-PANAS-SF; Thompson, 2007), which consists of a 10-item mood scale (Watson et al., 1988). In Study 1, respondents were asked to rate the extent to which Mark Smith’s answer made them feel five negatively valenced emotional adjectives (NA, e.g., frustrated, stressed, angry; *M* = 1.27; *SD* = 0.53; α = 0.86) and five positively valenced emotional adjectives (PA, e.g., enthusiastic, relaxed, happy; *M* = 3.36; *SD* = 0.87; α = 0.87) on a scale ranging from 1 (“not at all”) to 5 (“extremely”).

#### Attachment Orientations

Individuals’ attachment orientations were assessed using the adapted short form of Brennan et al. (1998) Experience in Close Relationships Scale (ECR), based on Fraley et al. (2015). This short form consists of nine items on two subscales measuring anxious attachment and avoidant attachment using a 7-point scale ranging from 1 (“strongly disagree”) to 7 (“strongly agree”). Anxious attachment (*M* = 2.83, *SD* = 1.57, α = 0.91) is comprised of three items (example item: “I often worry that other people do not really care for me”), and avoidant attachment (*M* = 3.45, *SD* = 1.32, α = 0.88) is comprised of six items (example item: “I don’t feel comfortable opening up to others”).

#### Personality

Personality traits can influence the perception of others (Judge et al., 2002). Hence, we controlled for personality traits as defined by the Big 5 personality traits, using the Mini IPIP scale (Goldberg et al., 2006). The five traits are Openness to Experience (*M* = 3.94, *SD* = 0.86, α = 0.79; example item: “I have a vivid imagination”), Conscientiousness (*M* = 3.90, *SD* = 0.84, α = 0.79; example item: “I get chores done right away”), Extraversion (*M* = 2.64, *SD* = 1.08, α = 0.87; example item: “I am the life of the party”), Agreeableness (*M* = 3.82, *SD* = 0.89, α = 0.85; example item: “I sympathize with others’ feelings”) and Neuroticism (*M* = 2.19, *SD* = 0.96, α = 0.82; example item: “I get upset easily”). Scores were reported on a 5-point scale ranging from (1) “very inaccurate” to (5) “very accurate” for all dimensions.

#### Conflict

We measured the expectation of conflict (α = 0.88) using an adapted intragroup conflict measure (Jehn and Mannix, 2001). Our adapted scale consists of four items and includes statements such as “I expect there to be _____ relationship tension working with Mark” and “I expect there to be _____ conflict of ideas working with Mark.” Participants indicated their agreement to these items on a 5-point scale ranging from (1) “No(ne)” to (5) “Extreme(ly).”

#### Likeability

Likeability was measured using one item, namely “I would like to work with Mark.” Participants indicated their agreement on a 5-point scale ranging from (1) “Strongly Disagree” to (5) “Strongly Agree.”

#### Demographics

McClure and Lydon (2014) found that anxious attachment was more strongly and reliably perceived in men rated by female participants, compared to women rated by male participants. Hence, we control for gender, in addition to age, work experience, and job level.

### Results

**FIGURE 1:**
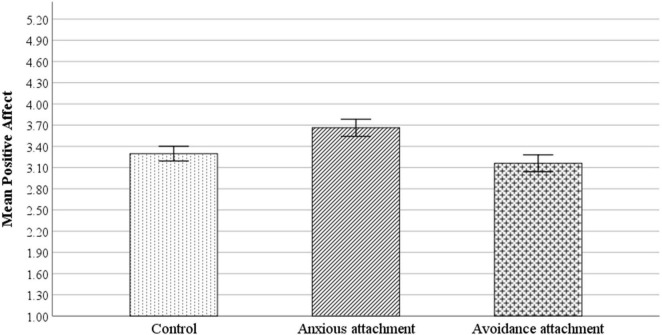
Main effect of anxious attachment on positive emotions compared to control and avoidance (*n* = 636).

Correlations of main variables are shown in Table 2. A simple ANOVA test (Figure 1) using condition (anxiety vs. avoidance vs. neutral) as the independent variable and positive affect as dependent variable, showed that reading the anxious attachment answer (*M* = 3.66, *SD* = 0.87) resulted in greater positive emotions, *F*(2,634) = 19.41, *p* < 0.01, *d* = 0.12 (see Figure 1), compared to those who read a neutral vignette (*M* = 3.29, *SD* = 0.78), and avoidance vignette (*M* = 3.16, *SD* = 0.89).

**TABLE 2:** Simple correlations between main variables (Study 1).

		*M*	*SD*	1	2	3	4	5	6	7	8	9	10
(1)	Avoidant attachment	3.45	1.32	(0.88)									
(2)	Anxious attachment	2.83	1.57	0.38**	(0.91)								
(3)	Openness to experience	3.94	0.86	−0.15**	−0.22**	(0.79)							
(4)	Conscientiousness	3.90	0.84	−0.17**	−0.40**	0.21**	(0.79)						
(5)	Extraversion	2.64	1.08	−0.42**	−0.22**	0.24**	0.12**	(0.87)					
(6)	Agreeableness	3.82	0.89	−0.53**	−0.19**	0.33**	0.19**	0.26**	(0.85)				
(7)	Neuroticism	2.19	0.96	0.30**	0.55**	−0.26**	−0.47**	−0.30**	−0.15**	(0.82)			
(8)	Negative affect	1.27	0.53	0.15**	0.25**	−0.13**	−0.33**	0.04	−0.21**	0.28**	(0.86)		
(9)	Positive affect	3.36	0.87	−0.21**	−0.14**	0.02	0.18**	0.15**	0.16**	−0.21**	−0.32**	(0.87)	
(10)	Likability	4.29	0.84	−0.16**	−0.10*	–0.02	0.18**	0.06	0.15**	−0.18**	−0.46**	0.61**	(0.88)
(11)	Conflict	1.42	0.61	0.10*	0.22**	−0.09*	−0.32**	0.07	−0.18**	0.25**	0.65**	−0.29**	−0.55**

Pearson correlations; Cronbach alphas in parentheses; **p < 0.01, *p < 0.05.

Specific *post hoc* comparisons (Bonferroni test) showed that in the anxious attachment condition participants reported significantly more positive affect compared to the control condition (*p* < 0.001, Δ*mean* = 0.366). Similarly, in the anxious attachment condition participants reported statistically significantly more positive affect compared to the avoidance attachment condition (*p* < 0.001, Δ*mean* = 0.502). Finally, there were no statistically significant differences in terms of positive affect between the control condition and the avoidance condition (*p* = 0.284, Δ*mean* = 0.136).

A similar pattern of findings is observed when testing the influence of attachment orientation (anxiety vs. avoidance vs. control) on conflict and likeability as dependent variables. Participants who were randomized to the anxious attachment condition (*M* = 4.48, *SD* = 0.76) indicated higher likability to work with the prototype colleague [i.e., Mark; *F*(2,634) = 43.03, *p* < 0.01, *d* = 0.17], compared to participants who read the control vignette (*M* = 4.12, *SD* = 0.88), or avoidant attachment vignette (*M* = 3.66, *SD* = 1.07). Specific *post hoc* comparisons (Bonferroni test) showed that in the anxious attachment condition participants reported more willingness to work with the prototype Mark compared to the control condition (*p* < 0.001), and compared to the avoidant attachment condition (*p* < 0.001). Finally, a statistically significant difference was observed comparing the willingness to work with the prototype colleague in the control condition vs. the avoidant condition (*p* < 0.001).

Concerning the effect of experimental condition on perceived potential conflict measure (alpha = 0.88), it was also found that reading the avoidant vignette (*M* = 1.78, *SD* = 0.81) resulted in more perceived potential conflict with Mark [*F*(2,634) = 22.34, *p* < 0.01, *d* = 0.13], compared to participants who read the control vignette (*M* = 1.50, *SD* = 0.63), or anxious prototype vignette (*M* = 1.34, *SD* = 0.57). *Post hoc* comparisons (Bonferroni test) showed that participants in the avoidant attachment condition expected more potential conflict, compared to the control condition (*p* < 0.001), and compared to the anxious attachment condition (*p* < 0.001). No difference for conflict were found comparing the control and the anxious conditions (*p* = 0.059).

Further analyses including covariates, revealed that the main effect of anxious attachment condition on positive affect remained statistically significant, *F*(1,634) = 20.43, *p* < 0.01, *d* = 0.42, after including negative affect, personality, self-reported attachment style of the participant, and demographics. From these covariates, negative affect [*F*(1,634) = 43.01, *p* < 0.01], avoidant attachment [*F*(1,634) = 4.86, *p* < 0.05], openness to experience [*F*(1,634) = 7.51, *p* < 0.01], extraversion [*F*(1,634) = 7.68, *p* < 0.01], and gender [*F*(1,634) = 7.18, *p* < 0.01] were statistically significantly related to positive affect.

Finally, we conducted a multivariate MANCOVA including positive affect and likeability as dependent variables, whereas condition (anxiety, avoidance, and control) remained as the sole fixed factor. Results showed that likeability was indeed predicted by condition, with the anxious attachment condition predicting greater degrees of likeability [*F*(1,408) = 43.02, *p* < 0.01, *d* = 0.52]. Importantly, the effect of the manipulation on positive affect remained significant [*F*(1,408) = 19.41, *p* < 0.01, *d* = 0.35]. Adding personality traits (Big-five) and self-reported attachment orientation of the respondent to the model did not change the main effect of the manipulation on both likability [*F*(1,408) = 38.84, *p* < 0.01], and positive affect [*F*(1,408) = 18.00, *p* < 0.01]. Additional effects are displayed in Table 3.

**TABLE 3:** Main effect of covariates (Study 1) for Conflict, Liking and Positive Affect as dependent variables.

	Conflict			Liking			Positive affect		
	*B*	*t*	*p*	*B*	*t*	*p*	*B*	*t*	*p*
Intercept	2.31	8.99	0.00	3.85	10.12	0.00	3.33	9.52	0.00
Avoidant attachment	–0.22	–0.38	0.70	–0.06	–1.81	0.07	–0.09	–2.63	0.01
Anxious attachment	0.16	0.28	0.78	0.02	0.63	0.53	0.008	0.30	0.77
Openness to experience	–0.01	–0.09	0.93	–0.15	–3.23	0.00	–0.09	–2.20	0.03
Conscientiousness	–0.23	–4.47	0.00	0.12	2.36	0.02	0.10	2.13	0.03
Extraversion	1.93	3.79	0.00	–0.01	–0.29	0.77	0.05	1.58	0.11
Agreeableness	–0.26	–4.60	0.00	0.11	2.13	0.03	0.05	1.12	0.26
Neuroticism	0.15	2.46	0.01	–0.11	–2.23	0.03	–0.10	–2.15	0.03
Condition (ANX)	–0.08	–1.85	0.07	0.78	8.80	0.00	0.46	5.64	0.00

*n = 636.*

## Study 2

### Sample and Procedure

The sample comprised 648 round-robin observations of 26 working professionals (50% female, *M*_*age*_ = 28.9 years; *SD* = 2.55 years) who provided weekly team member observations over a period of 12 weeks. Participants had an average of 6.31 years of work experience (minimum = 3 years, max. 16 years), and had no previous experience working together, organized in seven groups of three to four participants (two groups comprised of three members and five groups of four members). Informed consent was provided by all participants.

### Participants

All participants were part of an MBA program attending a course on consulting skills. Groups worked together during 12 consecutive weeks. They visited a real company and provided consultations during the duration of the course. Groups prepared company assessments, reported results to the client, and implemented some correcting strategies to aid the company in solving weaknesses observed during the consulting process. As a requisite, participants regularly met every week to coordinate their work and to plan further steps.

The data for this study were collected in two stages across a period of approximately 3 months. In the first stage, all participants received an electronic invitation to take part in the study, starting with an initial questionnaire including demographic questions (age, gender, tenure in their current job) and covariates. In the second stage of the study, participants received an email reminder every week for nine consecutive weeks. Some of the measures in the second stage were surveyed using a round-robin design, during which participants reported their affective experience and relationship-specific attachment toward each team member. Every week participants also reported how they think they had been making others feel in the group, using the same measure to report on their affective experience toward each group member. Each time, we also requested participants to report the date when the weekly group meeting took place. This was done to compute a measure of latency, calculated as the simple arithmetic difference between the date when the participant completed the questionnaire and when the meeting took place. This factor was included as a covariate in the analysis at a subsequent stage.

If a group missed or skipped a weekly meeting, we extended the period of assessment to 1 or 2 weeks. In that way, we reduced the attrition and maintained the same number of observations across groups. In total, we obtained *k* = 648 observations.

### Cross-Sectional Measures (Time 1)

Before starting the longitudinal section of the study participants completed two individual differences measures.

#### Emotional Contagion Scale

Participants completed the 18 items of the Emotional Contagion Scale (Hatfield et al., 1994). This scale evaluates the degree of susceptibility of individuals to catch other people’s emotions. It examines a person’s tendency to mimic others’ emotions. Participants were asked to evaluate the extent to which each of the items is true for them on a scale ranging from 1 (“not true for me”) to 4 (“always true for me”). Sample items included the following: “*When someone laughs hard, I laugh too*” and “*I’m very sensitive in picking up other people’s feelings*” (α = 0.70).

#### Short Form of the Positive and Negative Affect Schedule (I-PANAS-SF)

Positive and negative affect were measured using the Short Form of the Positive and Negative Affect Schedule (I-PANAS-SF; Thompson, 2007), which consists of a 10-item mood scale (Watson et al., 1988). Respondents were asked to indicate the extent to which they experience five negatively valenced emotional adjectives (NA, e.g., frustrated, stressed, and angry) and five positively valenced emotional adjectives (PA, e.g., enthusiastic, relaxed, and happy) on a scale ranging from 1 (“not at all”) to 5 (“extremely”).

#### Demographics

We control for gender, in addition to age, and job tenure.

### Longitudinal Measures

Participants completed the following scales every week for 9 weeks.

#### State-Affect Measure

Participants rated the extent to which each group member made them feel using five-positively valanced emotional adjectives (e.g., happy, relaxed, and confident; α = 0.84), and five-negatively valanced items (e.g., frustrated, angry, and stressed; α = 0.83). These items were adapted from Eisenkraft and Elfenbein (2010) and were all answered using a Likert-format scale, ranging from 1 (“completely disagree”) to 5 (“completely agree”).

#### Attachment Orientations

Anxious and avoidant attachment were measured using four items (Game, 2008), two for each attachment dimension. Participants were instructed to think about their current relationships with each team member and responded in terms of the extent to which they agreed with each of the items, ranging on a scale from 1 (“completely disagree”) to 5 (“completely agree”).

The avoidant attachment items measured the degree of discomfort with closeness (i.e., “I try to avoid having to go to this person for help or advice” and “I prefer to handle problems on my own rather than ask this person to help”; α = 0.88); whereas the anxious attachment items measure the degree of dependency (e.g., “I wish this person and I could be friends” and “I’d like to know more about this team member as a person”; α = 0.86). These four items were carefully selected and used for two main reasons. First, these four items were deemed to be the only items acceptable given that we were interested in measuring relationship-specific attachment toward individual team members (instead of employees’ supervisor) and the remaining items were not suited within this context (e.g., “I sometimes wonder whether I’m my supervisor’s favorite employee”). Second, given that we asked participants to complete these measures for every team member every week, the respective scales had to be short to retain participants. Hence, from an operational point of view, we decided to implement a shorter scale than the original scale (Game, 2011). Nonetheless, we obtained robust Cronbach alphas for each sub-scale (see above).

### Results

Variance and covariance of main variables are provided in Table 4.

**TABLE 4:** Variance-covariance of main variables (Study 2; *k* = 648 observations).

		*M*	*SD*	1	2	3	4	5	6	7	8
(1)	Anxiety attachment	3.86	0.86	0.75							
(2)	Avoidance attachment	2.37	0.98	–0.41	0.96						
(3)	Partner positive affect	3.49	0.65	0.28	–0.31	0.43					
(4)	Partner negative affect	1.74	0.66	–0.15	0.26	0.21	0.44				
(5)	Gender	−	−	–0.04	0.06	–0.04	0.05	0.25			
(6)	Age	29	2.60	–0.05	0.39	0.20	0.02	0.11	6.52		
(7)	Tenure	6.29	2.98	–0.06	0.42	–0.02	0.03	0.03	5.61	9.04	
(8)	Emotional contagion	2.87	0.29	0.05	–0.05	0.03	0.01	0.03	0.24	0.19	0.08

#### The Stability of Attachment-Related Orientations

In this subsection, we evaluate the stability of anxiety and avoidance attachment orientations over respective surveyed weeks. To do so, we decomposed the level-2 variance of attachment reports to determine whether significant stability emerges over time. A variance component was used to compute the variance-covariance matrix, whereas the estimator was a maximum likelihood. Random intercept and slope for time were considered; time was centered. Intra-class correlation (ρ) was the main estimator to determine the significance of variance at level-2 for the attachment-related measure.

Results revealed a significant amount of variance at level-2 (between-person variance) for anxious attachment, ρ = 0.42, *p* < 0.01. Similarly, a significant portion of variance at level-2 was found for avoidant attachment, ρ = 0.33, *p* < 0.01. These results indicate that it is reasonable to consider stable attachment orientations as emerging from the attachment self-reports obtained over time.

#### Dyadic Nature of Emotions Over Time

To appropriately capture the hypothesized effect, affect was measured using a round-robin design, resulting in a cross-classified data structure. To appropriately model the inherent dependency resulting from this design, an SRM (Kenny and Lavoie, 1984; Kenny, 1994) for indistinguishable dyads (i.e., members of each group cannot be distinguished from one another by some variable, such as gender) was used. This design allowed us to decompose the variance and determine the hypothesized associations.

The present study added a longitudinal design to the regular round-robin design used when implementing the SRM. As a result, we applied a social relations growth model (SRGM; Nestler et al., 2017). SRGM enables us to estimate the variability produced by actors, partners, groups, and dyads through time, and to assess which variables affect changes in attachment and affect over time.

In this analysis, time (measurement occasions), perceiver (a number to identify which participant was evaluating to his or her partner), target (a number to identify which partner was being judged by a certain actor), and dyad (a unique code identifying each pair of individuals evaluating each other), were modeled to produce nine variances estimates. We excluded the covariances of the model to allow the convergence of the model as we were not interested in examining those covariances.

We firstly ran an SRGM analysis without covariates. The timing variable was coded as zero for the first time and using integer numbers for the following measures (e.g., 1 = second measure, 2 = third measure, and so on). Secondly, in the case that the results of the first model suggested that there was significant variability in one of the components of the SRGM (dyad for the interest of the present research), we ran a second model which included the corresponding independent and dependent variables. We used unrestricted correlations to compute the variance-covariance matrix, whereas the estimator was restricted to maximum likelihood. Results of these analyses are presented in Tables 5, 6.

**TABLE 5:** Estimates of covariance parameters positive affect (Study 2).

Parameter		Estimate	Std. error	Wald *Z*	Sig.	95% confidence interval	
						Lower bound	Upper bound
Residual		0.21	0.02	13.29	0.00	0.18	0.24
Intercept + TimeR [subject = Actor]	Var(1)	0.11	0.05	2.15	0.03	0.04	0.27
	Var(2)	0.00	0.00	2.04	0.04	0.00	0.01
	Corr(2,1)	−0.59	0.24	−2.44	0.02	−0.88	−0.05
Intercept + TimeR [subject = Partner]	Var(1)	0.02	0.02	0.97	0.33	0.00	0.17
	Var(2)	0.00	0.00	0.30	0.77	0.00	0.06
	Corr(2,1)	1.00	0.00	−	−	−	−
Intercept + TimeR [subject = Dyad]	Var(1)	0.08	0.04	1.99	0.05	0.03	0.21
	Var(2)	0.00	0.00	0.10	0.92	0.00	17843.31
	Corr(2,1)	0.64	4.48	0.14	0.89	−1.00	1.00

*Random effect variances reported. Var(1): intercept of growth model; Var(2): growth curve function of the model; Corr(2,1): correlation between the intercept and the growth curve function; k = 648 observations.*

**TABLE 6:** Estimates of covariance parameters negative affect (Study 2; *k* = 648 observations).

Parameter		Estimate	Std. error	Wald *Z*	Sig.	95% confidence interval	
						Lower bound	Upper bound
Residual		0.20	0.01	13.49	0.000	0.17	0.23
Intercept + TimeR [subject = Actor]	Var(1)	0.15	0.06	2.29	0.02	0.06	0.35
	Var(2)	0.01	0.00	2.25	0.02	0.00	0.02
	Corr(2,1)	–0.41	0.25	–1.67	0.10	–0.77	0.14
Intercept + TimeR [subject = Partner]	Var(1)	0.04	0.09	0.40	0.69	0.00	4.85
	Var(2)	0.00	0.00	0.01	1.00	0.00	0.00
	Corr(2,1)	–1.00	121.95	–0.01	0.99	–1.00	1.00
Intercept + TimeR [subject = Dyad]	Var(1)	0.05	0.03	1.71	0.09	0.02	0.17
	Var(2)	0.00	0.00	0.59	0.56	0.00	0.01
	Corr(2,1)	–1.00	0.00				

*Random effect variances reported. Var(1), intercept of growth model; Var(2), growth curve function of the model; Corr(2,1), correlation between the intercept and the growth curve function; k = 648 observations.*

From the two models estimated (positive affect and negative affect), the variances for the perceiver were all significant. For positive affect, this means that people tended to report feeling more positive emotions toward the majority of their group members at the beginning of the study (σ_*A*0_0.11, *p* < 0.05), and this tendency remained mostly stable over time (σ_*A*1_0.004, *p* < 0.05).

Important for the present study are the dyadic effects. Observing a significant variance at the dyadic level for positive affect means that people tended to reciprocate their positive emotions during one-to-one interactions. Results at the dyadic level revealed a significant variance for positive affect in the very first one-to-one encounter reported between group members (σ_*D*0_0.077, *p* < 0.05). Conversely, people did not keep reciprocating positive dyadic emotions, with results showing that this effect disappeared over time (σ_*D*1_0.00, *p* > 0.05).

#### Effects of Anxious and Avoidant Attachment on Dyadic Positive Emotions Over Time

In the final stage of analyses, we tested the effect of anxious attachment and avoidant attachment on dyadic emotions over time. To appropriately model these analyses, we conducted a longitudinal random-effects model including positive and negative dyadic emotions as dependent variables and a between-person-centered version of attachment. As the model concerns a reciprocal effect, where anxious attachment should be associated with greater positive emotions reciprocated within dyads, we considered a cross-classified model including time and dyad as level-2 variables. A Bayesian estimator was chosen, including over 1,000 iterations. Results revealed that anxious individuals tended to elicit more positive one-to-one interactions, reciprocating positive emotions with each team member (β = 0.356, *p* < 0.01, *95%CI* [0.11/0.52]). Importantly, the effect of anxious attachment on dyadic positive emotions was significant only in initial interactions (σ_*D*0_0.078, *p* < 0.05), with no effect observed over time (σ_*D*1_0.00, *p* > 0.05), indicating that the positive impact that anxious participants have on others in one-to-one interactions was ephemeral.

Concerning avoidant attachment, we found the opposite effect, namely that avoidant individuals reciprocated fewer positive emotions during one-to-one interactions within groups (β = −0.272, *p* < 0.01, *95%CI* [−0.37/−0.17]). Contrasting these results with effects observed for anxious individuals, the effect reported for avoidant individuals remained stable over time, with significant variances during first encounters (σ_*D*0_0.033, *p* < 0.01), and steady negative effects over time (σ_*D*1_0.004, *p* > 0.01), emphasizing the chronicity of this attachment orientation.

We added several covariates to the model of anxious attachment predicting positive emotions. Adding emotional contagion, trait positive affect, trait negative affect, gender, or age into the previous model, did not change the observed effect of anxious attachment orientation on positive dyadic emotions. We also added an interaction between anxious attachment and avoidant attachment, but no effects were detected.

## Discussion

Extending previous research on attachment and early stages of close relationships (Brumbaugh and Fraley, 2010), the two presented studies analyzed the effect anxious and avoidant attachment had in perceptions of and actual reactions in initial interactions and over time in a working team context.

Using a round-robin design (Study 2), we found that in actual interactions with previously non-acquainted team members anxiously attached participants, make a positive first impression on others in initial team interactions. These initial reactions to anxious persons were corroborated in early perceptions of higher likeability, positive emotion and lower conflict toward descriptions of a potential job candidate that emulated the anxious attachment prototype (Study 1). The positive effect of employees’ anxious orientations may be due to a higher than usual degree of approach-behavior and closeness (expressed, for example, in the level of self-disclosure and interdependence with others). While anxious individuals are more likely to reduce distance from others and share personal experiences, vulnerabilities, and emotions as a form of interpersonal threat regulation (Mikulincer and Nachshon, 1991), such positive emotion-eliciting behavior also allows them to form a connection with others swiftly (Brumbaugh and Fraley, 2010). Over time, however, some of the more negative characteristics of anxious individuals (e.g., a negative view of self, strong dependency on others) become more apparent and might result in conflicts between interaction partners. Hence, it is likely that the attachment insecurity that drives anxious persons’ “pleasing others” behavior in initial interactions is the same factor that, over time, gradually comes to light and proves problematic for the relationship (Brumbaugh and Fraley, 2010).

Compared to anxious persons, avoidant individuals promote a distant and hence less positive image in both initial and longer-term interactions. Avoidant persons are also less likely to adopt an approach-orientated behavior in initial interactions unless triggered by the target team member (Spielmann et al., 2013). Indeed, partly as a strategy to preserve this distance, avoidant individuals are motivated to reduce unpleasantness in interactions overall (Brumbaugh and Fraley, 2010). This avoidance-related pattern is consistent across early and later stages of interacting in such working team settings and perhaps telling of the way the attachment system is behaviorally organized (Strand, 2020).

The results from the first study support assertions that patterns of attachment-related perceptions can arise under minimal interpersonal circumstances not necessarily directly related to close interpersonal relationships (Turan, 2016). The results from the second study further point to a continuity between interpersonal perception-behavior consistencies regarding the two insecure attachment patterns. Overall, the two studies point to links between attachment-distinctive interpersonal patterns of perception and behavior in interpersonal team interaction and resonate with discussions regarding perception-action links in adult attachment organization (Beckes and Coan, 2011).

The present research also speaks to potential partner dependency with regard to relationship initiation. McClure and Lydon (2014) stipulated that the attachment orientation of a potential partner, in our case working team members, could affect relationship initiation. To further examine such an effect, a longitudinal dyadic research design is needed, such as the one adopted in this study. On a more practical level, the present studies suggest greater attention should be devoted to better understanding how attachment-related orientations influence organizational outcomes and processes over time. For example, we would recommend teams and especially team leaders to implement activities to promote team member trust and psychological security, to buffer the potential negative effects of insecure attachment.

## Limitations and Future Research

Naturally, the current studies are not without limitations. We acknowledge that in the second study the team sample size is limited. However, participants in each team provided data points on weekly basis for up to four other team members, respectively. However, we acknowledge that the presented study should be viewed as exploratory, and we encourage future research to study team member interactions on a larger scale. It would be interesting for example, to see whether similar patterns of interpersonal perception and behavior across time are evident in virtual teams due to ease of data collection with the use of machine learning algorithms to study team member interactions online (Gruda and Ojo, 2021, 2022; Gruda et al., 2021, 2022).

In addition, identifying underpinning mechanisms that guide anxious individuals to behave in an apprehensive manner toward others over time would be of particular interest. That is because, although individuals exhibit a dominant attachment orientation, all individuals’ behavior is underpinned by both attachment orientations, and a combination thereof (Gruda and Kafetsios, 2020, 2022). Since even secure attached individuals would exhibit at least a small degree of anxious attachment, such findings would be beneficial to the understanding of behavior concerning relationship maintenance over time. In that regard, the study of relationships between team members or leader-follower relationships in particularly trying times – such as during a crisis – could also constitute an interesting setting to study the attachment perception-behavioral links (Gruda and Kafetsios, 2021). Finally, future studies could further investigate the relationship between relevant personality features (e.g., Big-5) and attachment orientations to provide a stronger explanatory model to predict interpersonal behaviors in work and other socially relevant contexts.

## Conclusion

In this manuscript, we examined the effect of attachment in team member interactions over time using an SRGM. We find that anxious attachment is, indeed, associated with eliciting positive emotions in others, especially compared to avoidant individuals. However, this effect seems to be short-lived and disappears over time, while in the case of avoidant individuals, the effect seems to be stable over time.

## Data Availability Statement

The raw data supporting the conclusions of this article can be made available by the authors, without undue reservation, to any qualified researcher.

## Ethics Statement

Ethical review and approval was not required for the study on human participants in accordance with the local legislation and institutional requirements. The patients/participants provided their written informed consent to participate in this study.

## Author Contributions

DG contributed to the conception and design of the study and wrote the first draft of the manuscript. RB performed the statistical analysis. KK and JM wrote sections of the manuscript. All authors contributed to manuscript revision, read, and approved the submitted version.

## Conflict of Interest

The authors declare that the research was conducted in the absence of any commercial or financial relationships that could be construed as a potential conflict of interest.

## Publisher’s Note

All claims expressed in this article are solely those of the authors and do not necessarily represent those of their affiliated organizations, or those of the publisher, the editors and the reviewers. Any product that may be evaluated in this article, or claim that may be made by its manufacturer, is not guaranteed or endorsed by the publisher.
